# Predictors of inappropriate antimicrobial prescription: Eight-year point prevalence surveys experience in a third level hospital in Spain

**DOI:** 10.3389/fphar.2022.1018158

**Published:** 2022-10-10

**Authors:** María Núñez-Núñez, Salvador Perez-Galera, José Antonio Girón-Ortega, Santiago Sandoval Fernández-Del-Castillo, Margarita Beltrán-García, Marina De Cueto, Ana Isabel Suárez-Barrenechea, Zaira R. Palacios-Baena, Pedro Terol-Barrero, Fernando Oltra-Hostalet, Ángel Arenzana-Seisdedos, Jesús Rodriguez-Baño, Pilar Retamar-Gentil

**Affiliations:** ^1^ Hospital Pharmacy Department, University Hospital Virgen Macarena, Seville, Spain; ^2^ Hospital Pharmacy Department, San Cecilio Clinical University Hospital, Granada, Spain; ^3^ Biosanitary Research Institute of Granada (Ibs.Granada), Granada, Spain; ^4^ Consortium for Biomedical Research in Epidemiology and Public Health (CIBERESP), Madrid, Spain; ^5^ Internal Medicine Department, University Hospital Virgen Macarena, Seville, Spain; ^6^ Institute of Biomedicine of Seville (IBiS) and CSIC, Seville, Spain; ^7^ Infectious Diseases and Microbiology Clinical Unit, University Hospital Virgen Macarena, Sevilla, Spain; ^8^ Department of Microbiology, University of Seville, Seville, Spain; ^9^ Consortium for Biomedical Research in Infectious Diseases (CIBERINFEC), Madrid, Spain; ^10^ Pediatric Department, University Hospital Virgen Macarena, Seville, Spain; ^11^ Emergency Unit, University Hospital Virgen Macarena, Seville, Spain; ^12^ Intensive Care Department, University Hospital Virgen Macarena, Seville, Spain; ^13^ Department of Medicine, University of Seville, Seville, Spain

**Keywords:** antimicrobial stewardship, point prevalence survey, quality assessment, antibiotic use, inappropriateness

## Abstract

Antibiotic stewardship programs (ASP) have already demonstrated clinical benefits. We aimed to describe the Point Prevalence Surveys (PPS) methodology implemented in our hospital as an efficient tool to guide ASP strategies. Annually repeated PPS were conducted from 2012 to 2019 at a 750-bed university hospital in South Spain. Key quality indicators and inappropriateness of antimicrobial treatment, defined strictly according to local guidelines, were described. Variables associated with inappropriate treatment were identified by bi/multivariable analysis. A total of 1,600 patients were included. We found that 49% of the prescriptions were inappropriate due to unnecessary treatment (14%), not first line drug recommended (14%), inadequate drug according to microbiological results (9%), unsuitable doses (8%), route (3%) or duration (7%). Samples collection presented a significant protective effect together with sepsis presentation at onset and intensive care unit admission. However, age, receiving an empirical treatment and an unknown or urinary source of the infections treated were independent risk factors for inappropriateness. Site and severity of infection were documented in medical charts by prescribers (75 and 61% respectively). PPS may allow identifying the main risk factors for inappropriateness. This simple methodology may be useful for ASP to select modifiable factors to be prioritized for targeted interventions.

## Introduction

Hospitals are efficient environments for the selection, amplification and dissemination of antimicrobial resistance, mostly due to the selection effect caused by high consumption of antimicrobial agents, and transmission of resistant pathogens. Inappropriate antimicrobial treatment is also associated with worse clinical outcomes, including mortality, in patients with severe infections. Consequently, improving the quality of antimicrobial prescribing should be the aim of any antimicrobial stewardship program (ASP) (Kollef et al., 1999; Patel et al., 2008; Powers, 2009; Retamar et al., 2013).

The evaluation of quality of antimicrobial prescriptions is a first step to design an ASP, as it provides information about the priority areas and needs for interventions (Dellit et al., 2007; Fishman, 2012; Rodríguez-Baño et al., 2012). Recognizing specific targets for improvement facilitates and optimizes the resources that should be invested in ASPs (Ramsay et al., 2003; Zarb et al., 2011; McGregor and Furuno, 2014). However, the best method for evaluating the quality of prescription is far from being well defined. While the evaluation method should be adapted to the aims and resources available at each centre (Fishman, 2012; Rodríguez-Baño et al., 2012), there is scarce information about how such evaluations should be developed.

Point prevalence surveys (PPS) have been proposed as an efficient approach to assess quality of prescriptions when resources are insufficient for continuous surveillance (Fishman, 2012); repeated PPS can also inform prescribing trends over time (Malcolm et al., 2013). Some experiences with the use of PPS have been published, including local and multicentre, even international surveys (Seaton et al., 2007; Ansari et al., 2009; Amadeo et al., 2010; Zarb and Goossens, 2011; Robert et al., 2012; Malcolm et al., 2013; Sinatra et al., 2013; Bozkurt et al., 2014; Pauwels et al., 2021). Some international initiatives to standardize the performance of PPS, as the European Surveillance of Antimicrobial Consumption (ESAC) (Ansari et al., 2009; Amadeo et al., 2010; Zarb and Goossens, 2011) or the Global-PPS (Pauwels et al., 2021), have been developed and some targets for quality improvements have been proposed (Zarb et al., 2011).

In this context, the objective of our study is to describe the PPS methodology implemented in our hospital to analyse modifiable predictors related to the quality of antibiotic usage. The methodology implemented may be useful to help with the design of future targeted interventions in ASPs.

## Methods

### Site and design

Annual PPS evaluating antimicrobial prescription appropriateness were conducted from 2012 to 2019 at a 750-bed university hospital in Seville, Spain. The PPS was performed every year during the last week of May. All patients with an antimicrobial prescription active at 8.00 a.m. on the day of the survey were evaluated; prophylaxis prescriptions were excluded for this analysis. Several wards were evaluated each day until the whole hospital was covered. The evaluations were performed by members of the local ASP team, including specialists in infectious diseases (ID), microbiology, internal medicine, intensive care, pediatrics, and hospital pharmacy. All the evaluators had been specifically trained and used the local antimicrobial guideline (www.http://www.hospital-macarena.com/antibioterapia/), which is updated at least every other year. A case report form was filled for each patient with an antibiotic prescription (Supplementary Figure S1), which was registered in an online database for further validation and analyses.

Antimicrobial stewardship activities have been performed in our center since 1997, including regular educational activities, elaboration of local guidelines, measuring of antibiotic consumption, and unsolicited advice for the management of patients with bacteremia and osteoarticular infections. A new, structured ASP was implemented in 2013, including specific objectives and indicators according to Spanish recommendations (Rodríguez-Baño et al., 2012) and included the formation of a multidisciplinary ASP team whose members meet every other week to evaluate the indicators and specific interventions to be added to those previously active.

The interventions added during the study period and performed by the ASP team were the post-prescription audits and *feedback* to prescribers of specific antibiotics (named here “high-impact antibiotics“) because of their ecological impact, toxicity, availability, or higher cost, including carbapenems, aztreonam, tigecycline, colistin, linezolid, daptomycin, and antifungals other than fluconazole; and for all antibiotic prescribed for ≥7 days, which were detected 3 days per week by consulting the electronic prescription system. In addition, specific activities were developed according to PPS results every year, and included mostly educational activities.

### Variables and definitions

Data from patients with antibiotic prescriptions were obtained from the clinical charts, and included: demographics, ward of admission, type of acquisition of infection (community-acquired, healthcare-associated), presentation with sepsis or septic shock, McCabe classification, source and etiology of infection, presence of bacteremia, type of antimicrobial use (empirical or targeted), prescribed drug(s), route, dose and duration in days. In addition, the following quality indicators were collected: whether the severity and source of the infection was specified in the chart or not (when not, the evaluators classified the severity and source according to other data in the chart); whether microbiological samples had been taken; whether the prescription was a monotherapy or combination; and duration ≤7 days. Prophylactic prescriptions were excluded.

The primary endpoint for the analyses was the adequacy of prescriptions, which were classified as “inappropriate” if the drug, route, dose and/or duration of the antibiotic prescription were incorrect according to the local guideline. Inappropriate prescriptions were sub-classified into “unnecessary” (UNN), “inadequate” drug (INA) according to susceptibility testing or lack of coverage for the main etiological pathogens for the specific syndrome, “adequate but not recommended” (ANR) if the drug chosen was adequate considering the spectrum, dose, route and duration but it was not the first option recommended by the guideline without any reason for not using the first option (this was considered a marker of the guideline adherence), “inadequate dose” (IDOS), “inadequate route” (IROU) and “inadequate duration” (IDUR). All doubtful cases were discussed within the evaluating team. In addition, 10% of the evaluation forms were reviewed by an external evaluator (PR).

### Statistical analysis

Qualitative and quantitative variables were described using absolute and relative frequencies, and median with interquartile range (IQR), respectively. The median was chosen over media for being less affected by outliers and because our data, such as age, presented a non-normal distribution of values. Missing data are shown. The association of factors related to inappropriate prescription was analyzed. The magnitude of the association was estimated by calculating the odds ratio with 95% confidence intervals (CI). Univariate analyses were performed using the chi-squared or Fisher’s exact test, and the Student’s t-test or Mann–Whitney U-test for comparison of categorical and continuous variables as appropriate, respectively. Multivariate analyses were performed by logistic regression; variables with a univariate *p* value < 0.15 were introduced, and selected using a backward stepwise manual procedure. All tests were performed using STATA 15.0.

## Results

### Patient‘s features and prescriptions

Overall, 1,600 patients with antimicrobial prescriptions were included in the annual PPS from 2012 to 2019. The median number of patients with antimicrobial prescriptions per study year was 201 (IQR, 180–223). The characteristics of the patients according to whether the prescription was classified as inappropriate or appropriate are shown in Table 1. The median age of patients with prescriptions was 69 years (IQR, 52–80 years) and 912 (57.2%) were male. The majority of patients were admitted in medical wards (941, 58.8%), had a non-fatal underlying condition (855, 61.1%) and not severe systemic inflammatory response syndrome at onset (884, 59.5%). Overall, 830 prescriptions (53.1%) were for community-acquired infections, and most were for empirical treatment (1,312, 82%); the most frequent sites of infections were the respiratory tract (514, 32.1%), intra-abdominal (326, 20.4%) and urinary tract infections (260, 16.3%). A more comprehensive categorization of the main reasons for inappropriateness according to the site of infections evaluated can be found in Supplementary Table S1.

**TABLE 1:** **Overall demographics, clinical features and antibiotics usage (N = 1600 antimicrobial prescriptions)**.

**Variables**		**Total** **N = 1600**	**Inappropriate N = 787**	**Appropriate N = 813**	***p* value**
		**N (%)**	**N (%)**	**N (%)**	
Gender N = 1595	Male	912 (57.00%)	426 (54.13%)	486 (59.78%)	**0.024**
	Female	683 (42.69%)	358 (45.49%)	325 (39.98%)	
Age N = 1600	Median (IQR)	69 (52-80)	71 (56-85)	65 (48-78)	**<0.001**
McCabe Score N = 1399	Nonfatal	855 (53.44%)	425 (54.00%)	430 (52.89%)	0.079
	Ultimately fatal	454 (28.38%)	248 (31.51%)	206 (25.34%)	
	Rapidly fatal	90 (5.63%)	39 (4.96%)	51 (6.27%)	
Type of department N = 1600	Emergency	149 (9.31%)	76 (9.66%)	73 (8.98%)	**<0.001**
	Medical	941 (58.81%)	504 (64.04%)	437 (53.75%)	
	Surgical	302 (18.88%)	160 (20.33%)	142 (17.47%)	
	Intensive Care	104 (6.50%)	22 (2.80%)	82 (10.09%)	
	Paediatrics	104 (6.50%)	25 (3.18%)	79 (9.72%)	
Healthcare-associated infections (HAIs) N = 1564	No	830 (51.88%)	422 (53.62%)	408 (50.18%)	0.083
	Yes	734 (45.88%)	341 (43.33%)	393 (48.34%)	
Sources (Site of infection) N = 1582	Respiratory	514 (32.13%)	247 (31.39%)	267 (32.84%)	**<0.001**
	Urinary	260 (16.25%)	137 (17.41%)	123 (15.13%)	
	Intra-abdominal	326 (20.38%)	161 (20.46%)	165 (20.30%)	
	Skin and soft-tissue infections and osteoarticular	232 (14.50%)	101 (12.83%)	131 (16.11%)	
	Endovascular & catheter	55 (3.44%)	13 (1.65%)	42 (5.17%)	
	Central Nervous System	12 (0.75%)	2 (0.25%)	10 (1.23%)	
	Not identified	115 (7.19%)	66 (8.39%)	49 (6.03%)	
	Others	68 (4.25%)	47 (5.97%)	21 (2.58%)	
Severity at presentation N = 1486	No	884 (55.25%)	492 (62.52%)	392 (48.22%)	**<0.001**
	Sepsis	438 (27.38%)	178 (22.62%)	260 (31.98%)	
	Septic shock	164 (10.25%)	62 (7.88%)	102 (12.55%)	
Site of infection described N = 1600	No	400 (25.00%)	236 (29.99%)	164 (20.17%)	**<0.001**
	Yes	1200 (75.00%)	551 (70.01%)	649 (79.83%)	
Severity described N = 1551	No	578 (36.13%)	315 (40.03%)	263 (32.35%)	**0.001**
	Yes	973 (60.81%)	446 (56.67%)	527 (64.82%)	
Samples taken N = 1600	No	657 (41.06%)	408 (51.84%)	249 (30.63%)	**<0.001**
	Yes	943 (58.94%)	379 (48.16%)	564 (69.37%)	
Empirical treatment N = 1594	No	288 (18.00%)	84 (10.67%)	204 (25.09%)	**<0.001**
	Yes	1312 (82.00%)	703 (89.33%)	609 (74.91%)	
High impact antibiotics N = 1594	No	1459 (91.19%)	729 (92.63%)	730 (89.79%)	0.085
	Yes	135 (8.44%)	57 (7.24%)	78 (9.59%)	
Combined medical treatments N = 1600	No	1295 (80.94%)	643 (81.7%)	652 (80.20%)	0.443
	Yes	305 (19.06%)	144 (18.30%)	161 (19.80%)	
Duration of treatment N = 1589	≤7 days	1280 (80.00%)	613 (77.89%)	667 (82.04%)	0.123
	8-14 days	251 (15.69%)	136 (17.28%)	115 (14.15%)	
	>14 days	58 (3.63%)	32 (4.07%)	26 (3.20%)	
Point Prevalence Survey (PPS) Year N = 1594	2012	199 (12.44%)	95 (12.07%)	104 (12.79%)	**<0.001**
	2013	232 (14.50%)	140 (17.79%)	92 (11.32%)	
	2014	204 (12.75%)	97 (12.33%)	107 (13.16%)	
	2015	224 (14.00%)	114 (14.49%)	110 (13.53%)	
	2016	221 (13.81%)	89 (11.31%)	132 (16.24%)	
	2017	185 (11.56%)	95 (12.07%)	90 (11.07%)	
	2018	182 (11.38%)	96 (12.20%)	86 (10.58%)	
	2019	153 (9.56%)	61 (7.71%)	92 (11.32%)	

Total = Total number of prescriptions evaluated; Inappropriate = if the drug, route, dose and/or duration of the antibiotic prescription were incorrect according to the local guideline; Appropriate = if the drug, route, dose and/or duration of the antibiotic prescription were correct according to the local guideline.

The most commonly used antimicrobial agents were amoxicillin-clavulanic acid (422, 26%), piperacillin-tazobactam (303, 19%), ceftriaxone (245, 15%), levofloxacin (169, 11%) and ciprofloxacin (89, 6%). Combination treatments and “high-impact antibiotics” drugs were prescribed in 311 (19%) and 135 (9%) patients respectively, and 1,280 (80%) of the prescriptions had a duration of ≤7 days.

### Prevalence and factors associated to inappropriate prescription. Quality indicators

**FIGURE 1:**
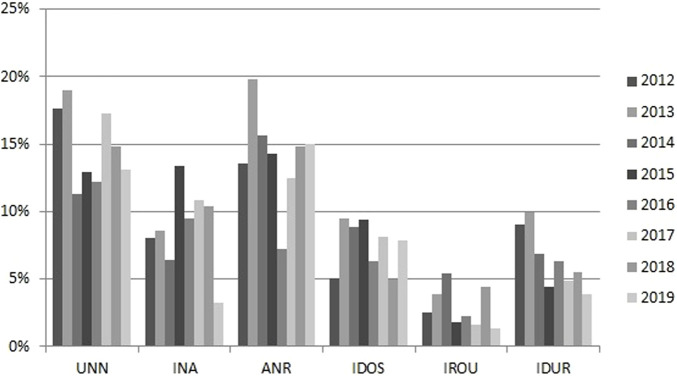
Specific reasons for which the antimicrobial prescriptions were evaluated as inappropriate per study year. Inappropriate prescriptions were sub-classified into “unnecessary” (UNN), “inadequate” drug (INA) according to susceptibility testing or lack of coverage for the main etiological pathogens for the specific syndrome, “adequate but not recommended” (ANR) if the drug chosen was adequate considering the spectrum, dose, route and duration but it was not the first option recommended by the guideline without any reason for not using the first option (this was considered a marker of the guideline adherence), “inadequate dose” (IDOS), “inadequate route” (IROU) and “inadequate duration” (IDUR).

Seven hundred eighty-seven (49.2%) prescriptions were evaluated as inappropriate using the very demanding criteria used, ranging over the years from 39.9 to 60.3%. The percentage of inappropriateness decreases over the study period; although we do not have enough points to estimate correctly a positive trend towards better antimicrobial usage. Being 2016 one of the years with the lowest percentage of inappropriate prescriptions. The cumulative prevalence of reasons (not mutually exclusive) was: UNN, 237 (14.8% of prescriptions); INA, 144 (9%); ANR, 226 (14.1%); IDOS, 121 (7.6%); IROU, 47 (2.9%); IDUR, 104 (6.5%). The evolution of the reasons for inappropriate prescription over the study years is shown in Figure 1. Among most frequently used drugs, levofloxacin followed by amoxicillin-clavulanate had the highest inappropriate rates (70 and 63% respectively).

The univariate and multivariate analysis of the association between different exposures and inappropriate prescription are shown in Table 2. Independent protective factors for inappropriate prescription were intensive care unit admission (adjusted OR [aOR] 0.48; 95% CI: 0.25–0.92); sepsis at presentation (aOR 0.68; 95% CI: 0.53–0.88); and having microbiological samples taken before treatment (aOR 0.57; 95% CI: 0.45–0.73); whereas age (aOR = 1.01; 95% CI: 1.01–1.02), empirical vs. targeted treatment (aOR = 1.86; 95% CI: 1.34–2.58); and unknown or urinary source of infection (aOR = 1.95; 95% CI: 1.22-3.12 and aOR = 1.48; 95% CI: 1.06-2.07 respectively) were independent risk factors for inappropriateness.

**TABLE 2:** Univariate and multivariate analysis of risk factors associated to inappropriate antimicrobial prescription.

Factors		OR crude (CI95%)	ORa (CI95%)
Gender	Male	ref	—
	Female	**1.26 (1.03–1.53)**	—
Age	Years	**1.05 (1.01–1.02)**	**1.01 (1.01–1.02)**
McCabe Score	Nonfatal	ref	—
	Ultimately fatal	1.22 (0.97–1.53)	—
	Rapidly fatal	0.77 (0.5–1.20)	—
Type of department	Emergency	ref	ref
	Medical	1.11 (0.78–1.57)	1.22 (0.84–1.76)
	Surgical	1.08 (0.73–1.60)	1.28 (0.82–1.98)
	Intensive Care	**0.26 (0.15–0.46)**	**0.48 (0.25–0.92)**
	Paediatrics	**0.30 (0.17–0.53)**	0.82 (0.40–1.71)
Healthcare-associated infections (HAIs)	No	ref	—
	Yes	0.84 (0.69–1.02)	—
Sources (Site of infection)	Respiratory	ref	ref
	Urinary	1.20 (0.89–1.62)	**1.48 (1.06–2.07)**
	Intra-abdominal	1.05 (0.80–1.39)	1.21 (0.89–1.65)
	Skin and soft-tissue infections and osteoarticular	0.83 (0.61–1.14)	0.89 (0.61–1.27)
	Endovascular/catheter	**0.34 (0.18–0.64)**	0.81 (0.40–1.66)
	Central Nervous System	**0.22 (0.05–0.99)**	0.55 (0.11–2.75)
	Not identified	1.45 (0.97–2.19)	**1.95 (1.22–3.12)**
	Others	2.42 (0.78–1.10)	**2.56 (1.39–4.71)**
Severity at presentation	No	ref	ref
	Sepsis	**0.55 (0.43–0.69)**	**0.68 (0.53–0.88)**
	Septic shock	**0.48 (0.34–0.68)**	0.77 (0.52–1.15)
Site of infection described	No	ref	ref
	Yes	**0.59 (0.47–0.74)**	0.82 (0.63–1.07)
Severity described	No	ref	ref
	Yes	**0.71 (0.58–0.87)**	0.92 (0.73–1.16)
Samples taken	No	ref	ref
	Yes	**0.41 (0.33–0.50)**	**0.57 (0.45–0.73)**
Empirical treatment	No	ref	ref
	Yes	**2.80 (2.13–3.70)**	**1.86 (1.34–2.58)**
High impact antibiotics	No	ref	—
	Yes	0.73 (0.51–1.05)	—
Combined medical treatments	No	ref	—
	Yes	0.88 (0.69–1.13)	—
Duration of treatment	≤7 days	ref	—
	8–14 days	1.29 (0.98–1.69)	—
	>14 days	1.34 (0.79–2.27)	—
Point Prevalence Survey Year (PPS)	Year	0.96 (0.91–1.00)	—

OR, odds ratio; CI, Confidence interval; ORa, Odds ratio adjusted by all categories: age; type of department; sources (site of infection); severity at onset; site of infection described; samples taken; empirical treatment.

Total = Total number of prescriptions evaluated; Inappropriate = if the drug, route, dose and/or duration of the antibiotic prescription were incorrect according to the local guideline; Appropriate = if the drug, route, dose and/or duration of the antibiotic prescription were correct according to the local guideline.

We have to highlight the implications of having a source of infection described in medical chart by the prescriber and those skin and soft-tissue infections and osteoarticular infections where both cases were identified as protective factors for unnecessary antimicrobial use (aOR = 0.44; 95% CI: 0.31-0.63 and aOR = 0.37; 95% CI: 0.18-0.76 respectively).

Regarding the quality indicators, the site of infection and severity were described in 400 (75%) and 973 (60.8%) of charts, respectively; microbiological samples had been taken in 943 (58.9%); combination treatment was used in 311 (19.4%), and duration was ≤7 days in 1,280 (80%). The evolution of the quality indicators rates over the years is shown in Table 3.

**TABLE 3:** Quality indicators rate per study year (2012–2019).

	Total N = 1,600	2012 N = 199	2013 N = 232	2014 N = 204	2015 N = 224	2016 N = 221	2017 N = 185	2018 N = 182	2019 N = 153
Site of infection described	1,200 (75%)	158 (79.4%)	191 (82.3%)	180 (88.2%)	164 (73.2%)	185 (83.7%)	116 (62.7%)	113 (62.1%)	93 (60.8%)
Severity described	973 (60.8%)	85 (42.7%)	151 (65.1%)	146 (71.6%)	117 (52.2%)	179 (81%)	102 (55.1%)	115 (63.2%)	78 (51.0%)
Microbiological samples taken	943 (58.9%)	97 (48.7%)	124 (53.5%)	122 (59.8%)	142 (63.4%)	121 (54.8%)	120 (64.9%)	110 (60.4%)	107 (69.9%)
Combination treatment	311 (19.4%)	43 (21.6%)	43 (18.5%)	43 (21.1%)	43 (19.2%)	38 (17.2%)	41 (22.2%)	28 (15.4%)	32 (20.1%)
Duration ≤7 days	1,280 (80%)	144 (72.4%)	180 (77.6%)	166 (81.4%)	189 (84.4%)	181 (81.9%)	150 (81.1%)	141 (77.5%)	129 (84.3%)

N(%), number of patients (percentage).

## Discussion

In this study, we present the experience in our center performing yearly PPS throughout 8 years to evaluate quality of antimicrobial prescriptions. Treatments were assessed as inappropriate if the drug, route, dose, and/or duration of the antibiotic prescription were incorrect according to the local guideline and the criteria were not modified throughout the years. Using these highly demanding criteria including aspects in different domains, almost half of the prescriptions were classified as inappropriate, and several variables were found to be associated with inappropriate prescriptions.

PPS, as all prevalence studies, has advantages and disadvantages. Our experience shows that PPS can efficiently provide relevant information for the development of specific interventions in our ASP. Although several experiences in the use of PPS to evaluate quality of antimicrobial prescription have been reported in the literature, detailed information about the specific methodology used, beyond ESAC protocols (Ansari et al., 2009; Amadeo et al., 2010; Zarb and Goossens, 2011), is scarce. We designed a collection data form aimed at identifying wards, profile of prescriptions and patients, and basic quality indicators that would help in understanding the domains in which improvement were needed, and the priority areas to intervene. Also, the data collection was designed considering the needs for simplicity and trying to avoid subjectivity. This approach allowed us to evaluate not only the overall inappropriateness but also, reasons for inappropriateness, prevalence for some quality indicators and factors associated with inappropriate prescriptions.

In the present situation, we consider that ASP must be highly demanding when evaluating antimicrobial prescriptions. While in the past some aspects such as dosing, selection of the drugs or duration of treatment were less specific, we decided to be very strict in evaluating the adherence to the local guidelines. Obviously, not all reasons for inappropriateness are equally important, and our guidelines is more “permissive” for the use of broad spectrum drugs in patients with severe infections or admitted to the ICU, and much less for infections in which targeted interventions were prioritized such as urinary tract infections or community-acquired pneumonia; our guidelines is also very strict in avoiding combination therapy except in specific situations. Therefore, the interpretation of the data must be tailored by the ASP team. The high proportion of inappropriate treatments found is not comparable to data from other studies but is useful for the decisions about interventions. While some previous studies only considered the microbiology susceptibility for assessment of appropriate prescriptions (Davey and Marwick, 2008), others considered guideline compliance (Seaton et al., 2007; Ansari et al., 2009; Amadeo et al., 2010; Zarb and Goossens, 2011; Robert et al., 2012; Bozkurt et al., 2014; Xie et al., 2015); however, guidelines may be more or less strict in their recommendation. Such differences should be taken into account for the generalizability of the interventions published (Retamar et al., 2013; Spivak et al., 2016; Magill et al., 2021).

The intrinsic limitations of PPS must be considered. The information collected in over a week may not be representative of all prescriptions, and seasonality is not considered. However, by being repeated annually, we can evaluate trends. Also, longer prescriptions, which were more probably inappropriate, are overrepresented with point prevalence designs. To avoid usual problems with PPS, we were careful to assess that exposures to predictors for inappropriate prescription occurred before, not after the prescription. Also, specific potential sources of bias in our evaluations were considered. In order to reduce inter-evaluator variability, they were all members of the ASP team and were trained in the criteria interpretation; doubtful cases were openly discussed for group agreement, and a subset of cases was reviewed.

Regarding the criteria used to evaluate the prescriptioins, Kunin et al. (Kunin et al., 1973) developed a set for classifying prescriptions as appropriate or probably appropriate, which were used in other studies (Gyssens et al., 1992; Apisarnthanarak et al., 2006). This classification included different scenarios: an antibiotic is needed, no other drug is preferred, and there are no deficiencies in dosing or duration. DePestel et al. compared four different criteria to evaluate antibiotic appropriateness: fixed predefined local definitions, microbiology results, review of medical records and ID physician evaluation using Kunin’s criteria (DePestel et al., 2014); the appropriateness assessed by an ID differed significantly compared with other definitions, and tended to classify more prescriptions as inappropriate. A meta-analysis of ASP interventions suggest that the indication, the choice of drug, the route, the dosage, the frequency and the duration of administration have to be properly determined for performing an evaluation (Davey et al., 2005). We adapted the criteria by Kunin et al. to our local guideline; we also added the criteria “adequate, not-recommended” when the use of a drug was acceptable but not the first choice in our guidelines, which was the second cause of inappropriateness in our study. This allowed us to evaluate the adherence to the local guidelines for the preferred drugs. Magill et al. followed a similar approach in their cross-sectional study in 192 in US hospitals. Their definition of “unsupported antimicrobial” included unnecessary antimicrobials, deviations from recommended guidelines or excessive duration. They also found that half of the patients audited had an “unsupported” antimicrobial prescription (Magill et al., 2021).

The type of department where patients were admitted was collected. This information would allow ASP team to plan and coordinate different interventions according to specific targets in each area. In that sense we recognized Internal Medicine, General Surgery, Intensive Care Unit and Emergency as the main prescribers in our center so continuous and specific intervention could be implemented in these departments.

Throughout the 8 years, empiric treatments represented more than 40% of the prescriptions evaluated; educational efforts were made over the years trying to improve microbiological diagnosis, allowing targeted treatments; an increasing trend was observed for this indicator. Regarding antimicrobial tests, notice that in 2016 we moved from CLSI to EUCAST guidelines in the interpretation of antimicrobial susceptibility. The potential changes in our local guidelines and moreover, the dissemination of this information to prescribers may influence that 2016 was the year with a lower percentage of inappropriate prescriptions. In contrast, the description of the source of infection in medical charts by prescribers, which was identified as a protective factor for unnecessary prescriptions, decreased over the study period. This information informed us about the necessity of enhancing this aspect through targeted educational activities. ASP also played an important and maintained positive role in the management of osteoarticular infections, being identified as a protective factor for unnecessary treatment. Although, targeted interventions to improve female UTIs seem to be required.

In our study, amoxicillin-clavulanate was the most common drug used, which had a high rate of inappropriateness. This was also observed in other PPS studies (Seaton et al., 2007; Ansari et al., 2009; Amadeo et al., 2010; Zarb and Goossens, 2011; Cooke et al., 2014). Amoxicillin-clavulanate had been recommended as first choice to treat many empirical syndromes in our previous guidelines (urinary, respiratory, intraabdominal and soft tissue infections); as a response to the PPS results and to the fact that susceptibility to this antibiotic among *Escherichia coli* was decreasing in Spain (Ortega et al., 2012; Rodríguez-Baño et al., 2013), we changed the recommendation in some syndromes. A similar phenomenon is being observed regarding *Pseudomonas aeruginosa* susceptibility to piperacillin-tazobactam, the second most prescribed antimicrobial. Of note, the low prevalence of overall prescriptions, and specifically, of carbapenems, cephalosporins, and fluoroquinolones, is probably as a consequence of the long tradition of stewardship activities in our hospital.

As conclusion, in our experience, repeated PPS provided efficient and useful information for the design of ASP interventions; consensus for the criteria defining inappropriate use, aiming at identifying areas of improvement, is needed.

## Data Availability

The raw data supporting the conclusions of this article will be made available by the authors, without undue reservation.
