# Supplementary material for: Predictors of inappropriate antimicrobial prescription: Eight-year point prevalence surveys experience in a third level hospital in Spain
Source: Front Pharmacol. 2022 Oct 10;13:1018158. doi: 10.3389/fphar.2022.1018158 (PMC9592087; doi:10.3389/fphar.2022.1018158)
Supplement: Supplementary file 1 [file Table1.DOCX]

**Supplementary table 1. Main reasons for inappropriateness according to site of infection**

|  | **Unnecessary antibiotic**  N=237 | **Adequate but non-recommended** N=226 | **Inactive antibiotic** N=144 | **Inadequate dose** N=121 | **Inadequate administration route** N=47 | **Inadequate duration** N=104 |
| --- | --- | --- | --- | --- | --- | --- |
| **Site of infection** | **N (%)** | **N (%)** | **N (%)** | **N (%)** | **N (%)** | **N (%)** |
| Respiratory | 64 (27.0%) | 55 (24.3%) | 43 (29.9%) | 62 (51.2%) | 16 (34.4%) | 34 (32.7%) |
| Urinary | 31 (13.1%) | 59 (26.1%) | 29 (20.1%) | 20 (16.5%) | 11 (23.4%) | 13 (12.5%) |
| Intra-abdominal | 44 (18.6%) | 58 (25.7%) | 26 (18.1%) | 16 (13.2%) | 8 (17.2%) | 18 (17.3%) |
| Skin Soft Tissue & osteoarticular | 10 (4.2%) | 29 (12.8%) | 31 (21.5%) | 11 (9.1%) | 7 (14.9%) | 25 (24.0%) |
| Endovascular/catheter | 2 (0.8%) | 3 (1.3%) | 1 (0.7%) | 1 (0.8%) | 0 (0%) | 5 (4.8%) |
| Central Nervous System | 1 (0.4%) | 0 | 1 (0.7%) | 1 (0.8%) | 0 | 0 |
| Others/Not identified | 85 (35.9%) | 22 (9.7%) | 2 (1.4%) | 10 (8.3%) | 5 (10.6%) | 9 (8.7%) |
