# Supplementary material for: Predictors of inappropriate antimicrobial prescription: Eight-year point prevalence surveys experience in a third level hospital in Spain
Source: Front Pharmacol. 2022 Oct 10;13:1018158. doi: 10.3389/fphar.2022.1018158 (PMC9592087; doi:10.3389/fphar.2022.1018158)
Supplement: Supplementary file 2 [file DataSheet1.docx]

**Supplementary figure 1. Patient-level evaluation form for yearly Point Prevalence Surveys (PPS)**

| **POINT PREVALENCE SURVEYS FOR EVALUATING THE QUALITY OF ANTIMICROBIAL PRESCRIBING.** |
| --- |
| **Patient:** ….................................. **Age:** .... **Gender:** male/female **Department:** ……………………………….   - **Reason for prescribing:** prophylaxis (surgical/other), empirical treatment, targeted treatment. - **Acquisition:** community-acquired, health care-associated, nosocomial. - **Severity:**   - Indicated by the prescriber: yes/no.   - Index: non sepsis, sepsis, severe sepsis and septic shock. - **McCabe classification:** non-fatal, ultimately fatal, and rapidly fatal. - **Special situations:** neutropenia (yes/no), allergies (yes/no), other - **Source:**    - Indicated by prescriber, not indicated and determinate in evaluation. - Central nervous system, otolaryngology, upper respiratory tract, pneumonia, ventilator associated pneumonia, decompensated COPD, other respiratory, endocarditis/vascular, catheter-related, intrabdominal, bile, gastroenteritis, urinary, genital, osteoarticular, soft tissue, without focused, others. - Bacteraemia, surgical wound infection, device associated infection. - **Take sample:** yes/no - **Etiology and most important resistance profile: …** - **Antibiotics prescribed and assessment:** only active antibiotics.  \| Nº \| Antibiotic \| Route \| Dose \| Duration \| Appropriate \| Inappropriate \| \| --- \| --- \| --- \| --- \| --- \| --- \| --- \| \| 1 \|  \|  \|  \|  \|  \|  \| \| 2 \|  \|  \|  \|  \|  \|  \| \| 3 \|  \|  \|  \|  \|  \|  \| \| 4 \|  \|  \|  \|  \|  \|  \|  \| Nº \| Reasons for inappropriateness \| Reasons for inappropriateness \| Reasons for inappropriateness \| \| --- \| --- \| --- \| --- \| \| 1 \|  \|  \|  \| \| 2 \|  \|  \|  \| \| 3 \|  \|  \|  \| \| 4 \|  \|  \|  \|   * AB-ANR (Adequate non-recommended)**,** AB-UNN (unnecessary antibiotic)**,** AB-IN (invalid antibiotic), DOS (inappropriate dose), ROU (inappropriate route) and DUR (inappropriate duration).  **INSTRUCTION SHEET:**  **Prescription indication:**  If the patient presents several antibiotics at the same time and the reasons are different (ie fosfomycin for urinary tract infection and cefazolin for surgical prophylaxis, there will be a separate tab for each antibiotic.  - **Prophylaxis (Surgical/Other):** including "covert prophylaxis", ie persistence of antibiotic after surgery without symptoms of infection. If appear fever or other, it must be considered an empirical treatment.  - **Empirical treatment:** prior to know microbiological isolation and sensitivity.  - **Targeted treatment:** when the antibiogram is available.  **Acquisition:**  - **Community acquired:** less than 48 hours from admission.  - **Health care-related:** one of: admission with more than 48 hours in the prior year, outpatient with intravenous treatment or in haemodialysis unit or in day hospital, diagnostic or therapeutic procedure performed on an outpatient in the 30 days before the episode, nursing home and long-stay centres.  - **Nosocomial:** more than 48 hours of admission or less than 7 days from discharged.  **Severity:**  - **Indicated by prescriber:** any data collected in history.  - **No sepsis:** no criteria for sepsis.  - **Sepsis:** 2 criteria: fever (>38°C) or hypothermia, tachypnea (>20 breaths per minute) or PCO2 <32 mmHg, tachycardia (>90 bpm), leucocytosis (>12.000 cells/cc) or leukopenia (<4.000 cells/ml) or left shift neutrophil (immature neutrophil count in peripheral blood >10%).  - **Severe sepsis/shock Sepsis:** sepsis with data from tissue/organ dysfunction, hypoperfusion (hypotension, acidosis, lethargy...).  **McCabe classification:**  **- Non fatal (NF):** underlying disease but without death provided for at least five years.  **- Ultimately fatal (UF):** predictable death due to underlying disease in <5 years.  **- Rapidly fatal (RF):** predictable death due to underlying disease in <3 months.  **Source:**  - **Indicated** by prescriber or not specified and determined in the evaluation.  - **Without focus/unknown:** if no specification in the history regarding the source of infection  - **Others**: bacteraemia, surgical infection, device related infection (specify: urinary catheter, catheter, pacemakers, prosthetic joint, valve...).  **Etiology and more important resistance patterns:**  - Full name and most significant antimicrobial resistance.  **Prescribed antibiotics and inappropriate measurement:**  Gold standard: local antimicrobial guideline (<http://www.hospital-macarena.com/antibioterapia/>)  **Reasons of inappropriateness:**  - **ATB-UNN (unnecessary antibiotic):** the patient has no clinical infection  - **ATB-INA (non-active antibiotic):** if prophylaxis or empirical treatment and the antimicrobial does not appear in the guide as recommended for the syndrome and no microbiological activity on the most probably microorganisms (eg. Vancomycin for urinary tract infections). If targeted therapy appears as “resistant” in susceptibility test.  - **ATB-ANR (adequate not-recommended):** if the drug was correct considering the spectrum, dose, route and duration but it was not the option recommended by the local guideline (this is considered a marker of the guideline adherence) ie. Meropenem for community-acquired pneumonia. If targeted treatment it appears as sensitive but the spectrum is excessive (ie. Meropenem for *Escherichia coli* sensitive to amoxicillin).  - **DOS (inadequate dose):** dose references according to Guidelines. Consider individual dose adjustments according to liver and kidney function.  - **ROUTE (inadequate route):** if the third day of treatment the symptoms are controlled (hemodynamic stability, decrease fever and inflammatory markers) and patient has oral route available, the intravenous treatment is considered inappropriate.  - **DUR (excessive length):** duration references according to Guidelines.  *With one reason of inappropriateness, the overall prescription will be considered inappropriate. |
